# Supplementary material for: Mcph1, mutated in primary microcephaly, is also crucial for erythropoiesis
Source: EMBO Rep. 2024 Apr 11;25(5):19. doi: 10.1038/s44319-024-00123-8 (PMC11094029; doi:10.1038/s44319-024-00123-8)
Supplement: Supplementary file 1 — Appendix [file 44319_2024_123_MOESM1_ESM.pdf]

Table of Contents

Appendix Figure S1 page 2

Appendix Figure S2 page 3

Appendix Figure S3 page 4

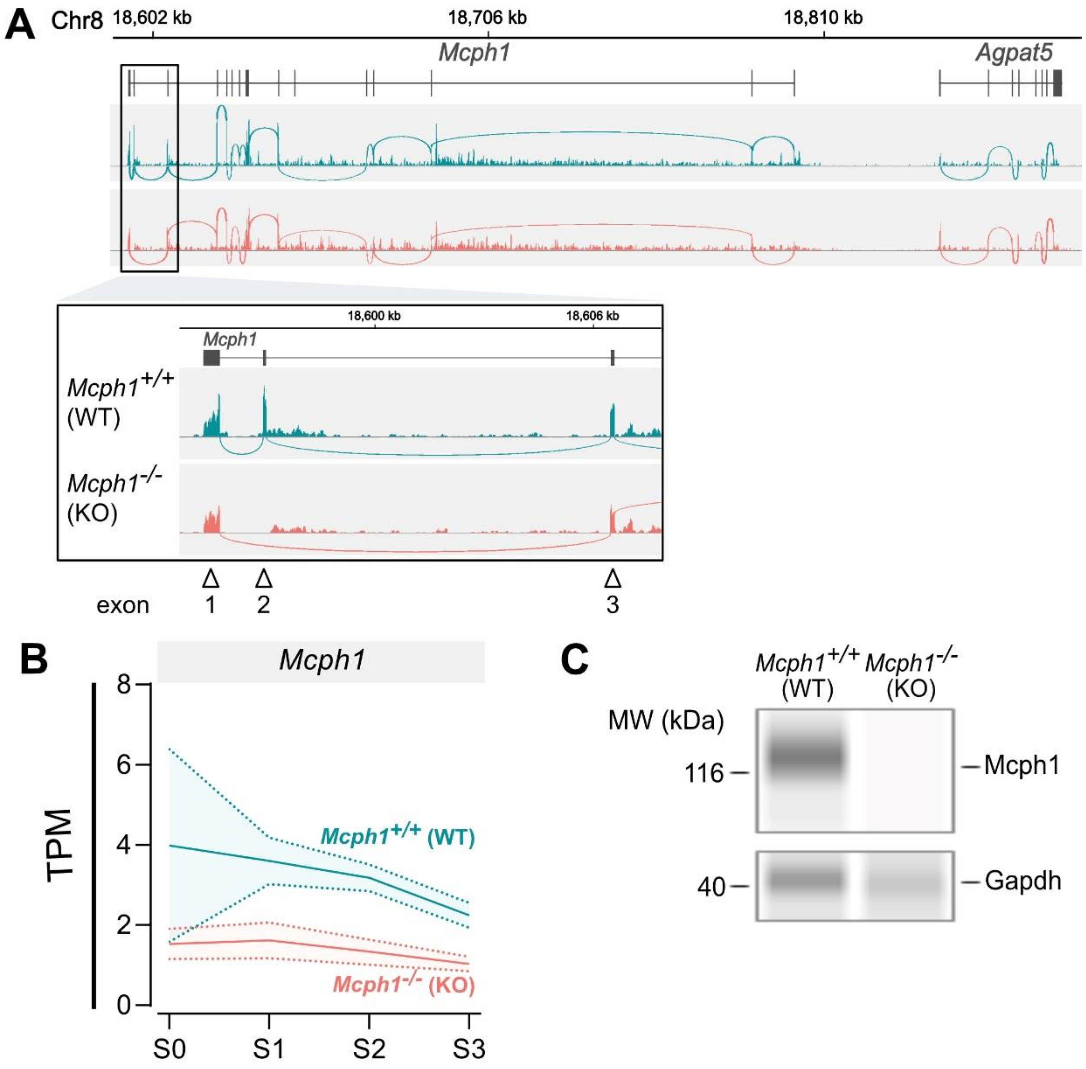

Appendix Figure S1: Characterization of *Mcph1* knockout mouse model. (A) Sashimi plot generated from aligned RNA-Seq data of the S3 subpopulation of WT (blue) and KO (red) mice at the *Mcph1* locus. Exons are represented by a gray box and introns by gray lines. Depth of coverage is represented by a histogram and reads supporting a junction by colored lines. Zooming in on exons 1, 2 and 3 indicated by triangles shows the splicing of these exons in fetal liver cells of *Mcph1*$^{+/+}$ (blue) and *Mcph1*$^{-/-}$ (red) mice. (B) Expression data of *Mcph1* during erythroid differentiation. Data expressed in transcript per million (TPM). Mean ± SD (filled area) of 2 experiments. (C) Simple Western for Mcph1 in protein extract from mouse fetal liver at E12.5.

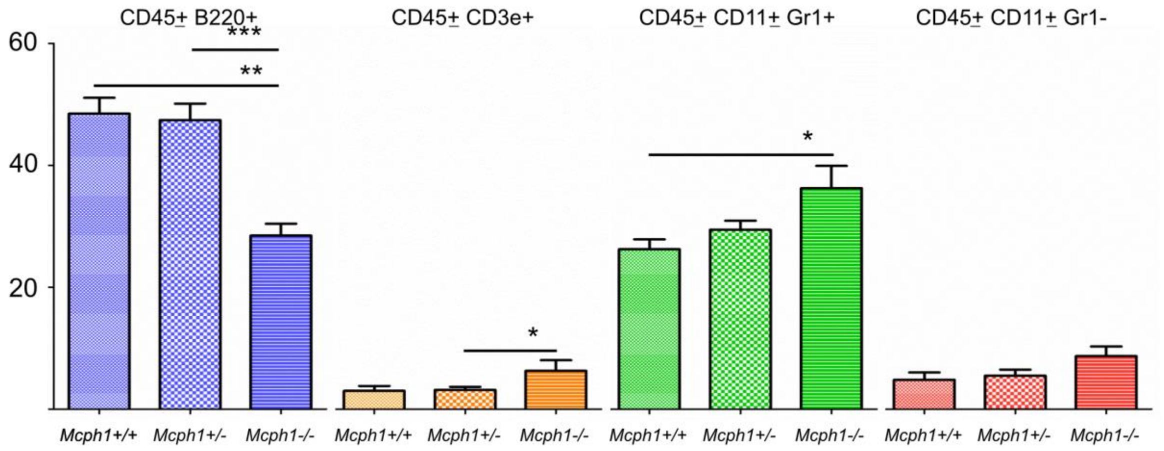

Appendix Figure S2: Hematopoiesis in liver at birth. Liver from Wild type (WT, *Mcph1*$^{+/+}$) and knockout (KO, *Mcph1*$^{-/-}$) mice were collected at birth (day 0). The proportion of B lymphocyte (CD45^{+}B220^{+}), T lymphocyte (CD45^{+}CD3e^{+}) and myeloid cells (CD45^{+}CD11b^{+}Gr1^{+}; CD45^{+}CD11b^{+}Gr1^{-}) were estimated.

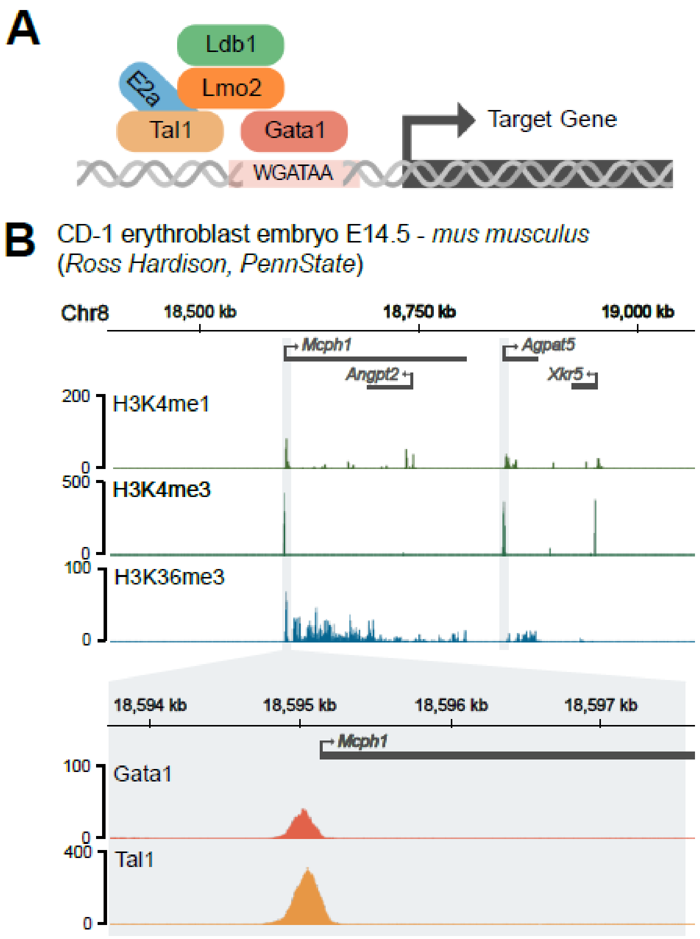

Appendix Figure S3: Gata1 and Tal1, master regulators of erythropoiesis, bind to the promoter region upstream of *Mcph1*. (A) Schematic showing the binding of the major complex controlling terminal erythroid differentiation. The binding of Gata1 (red) to the specific WGATAA motif and Tal1 (orange) at the promoter of the target gene allows the formation of the Gata1-Tal1-Ldb1 complex and activates the transcription of the target gene. (B) Chip-Seq gene track of the *Mcph1* locus (ENCODE project, ENCDO083AAA, Ross Hardison, PennState) on CD-1 cells (Ter119$^+$ erythroblasts) isolated from fetal mouse liver after 14.5 days of development (E14.5). Transcription start site and gene orientation are indicated by an arrow. Putative promoters (gray) are highlighted by the presence of H3K4me1 (green) and H3K4me3 (green) signals. Transcriptionally accessible genes are highlighted by the presence of an H3K36me3 signal (blue). Putative Gata1 (red) and Tal1 (orange) binding sites are revealed at the *Mcph1* promoter by Chip-Seq experiments. p-values of signals.
