# Supplementary figures and images for: Mcph1, mutated in primary microcephaly, is also crucial for erythropoiesis
Source: EMBO Rep. 2024 Apr 11;25(5):19. doi: 10.1038/s44319-024-00123-8 (PMC11094029; doi:10.1038/s44319-024-00123-8)

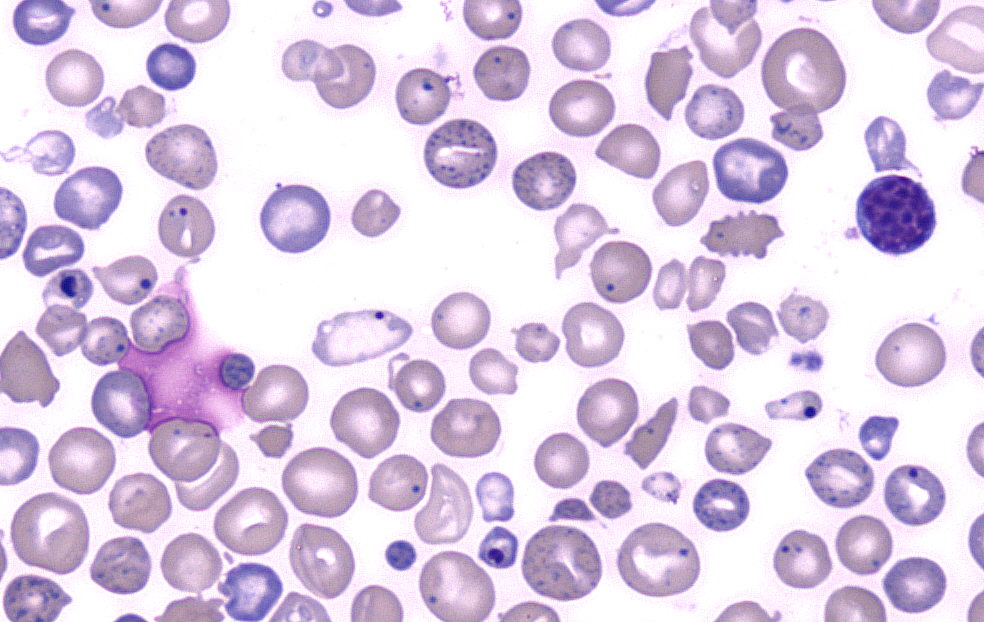

Supplement: Supplementary file 2 — Source data Fig. 1 [file 44319_2024_123_MOESM2_ESM.zip › Figure 1/1D/139-119614(KOBloodSmear).JPG]

# KO7-WT4 actine

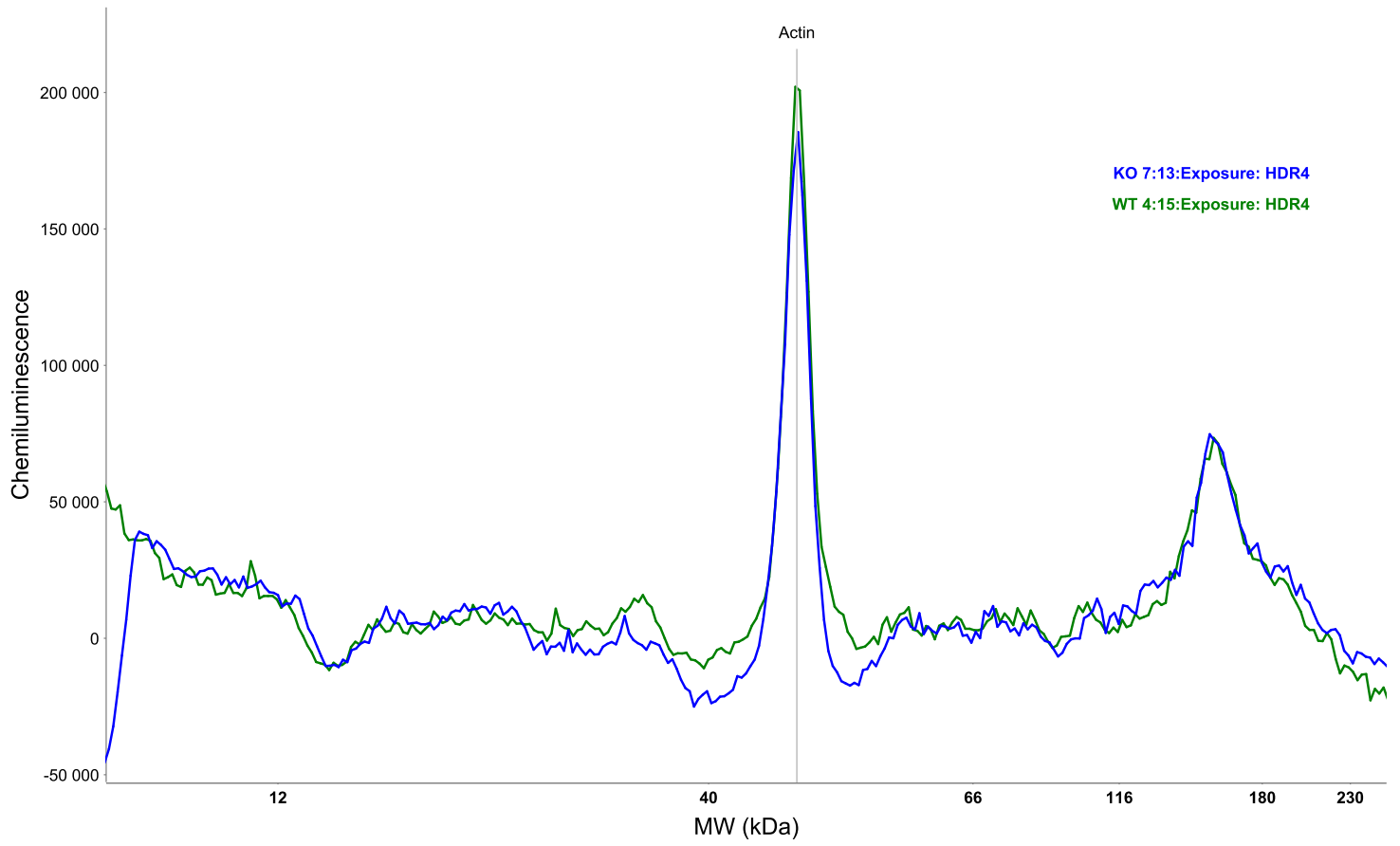

Supplement: Supplementary file 4 — Source data Fig. 3 [file 44319_2024_123_MOESM4_ESM.zip › Figure 3/3G/2021-07-13_14-16-52_2021-07-12_KO7-WT4 actine.pdf]

# KO7-WT4 P21 MCPH1

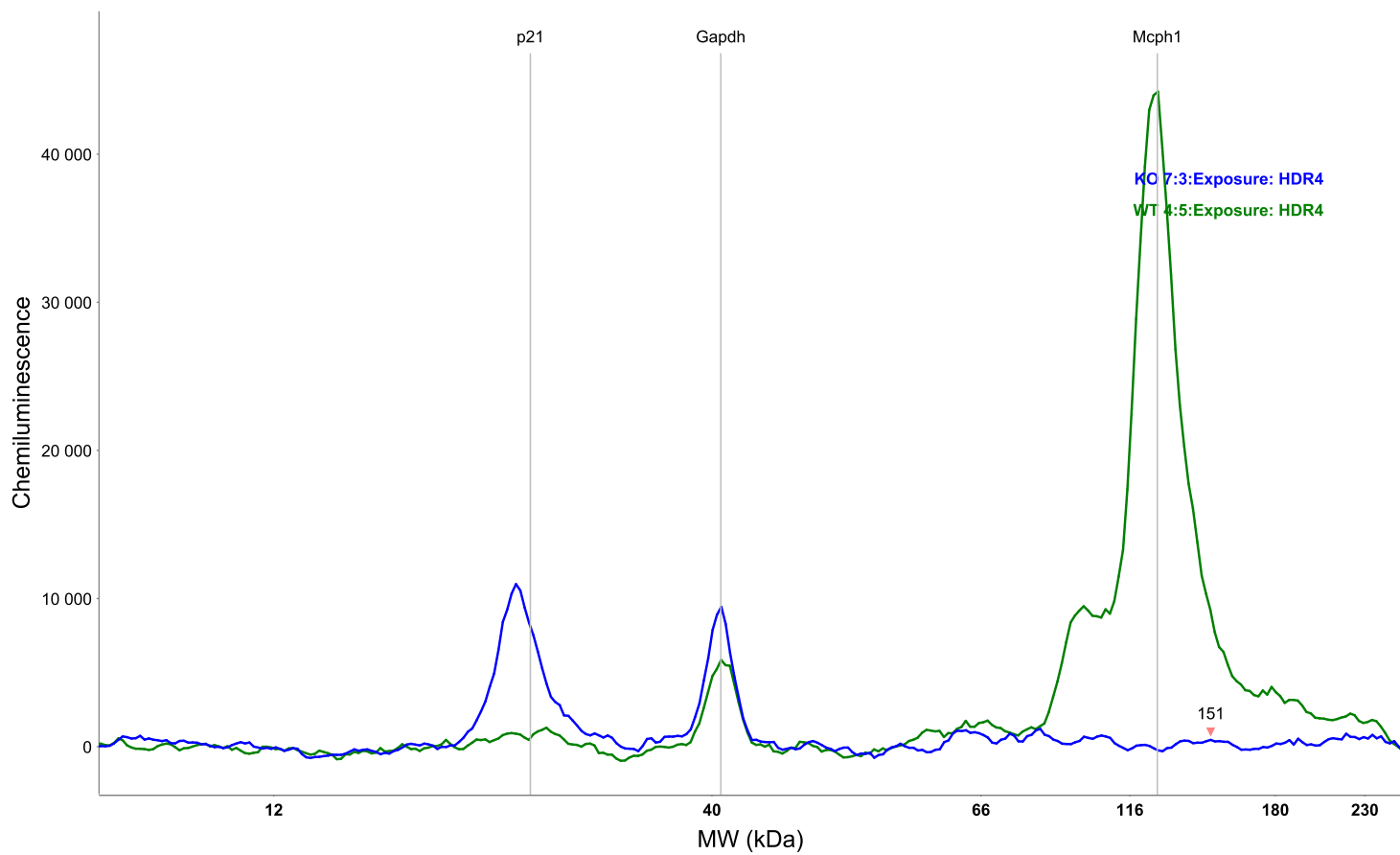

Supplement: Supplementary file 4 — Source data Fig. 3 [file 44319_2024_123_MOESM4_ESM.zip › Figure 3/3G/2021-07-13_14-16-52_2021-07-12_KO7-WT4 P21 MCPH1.pdf]

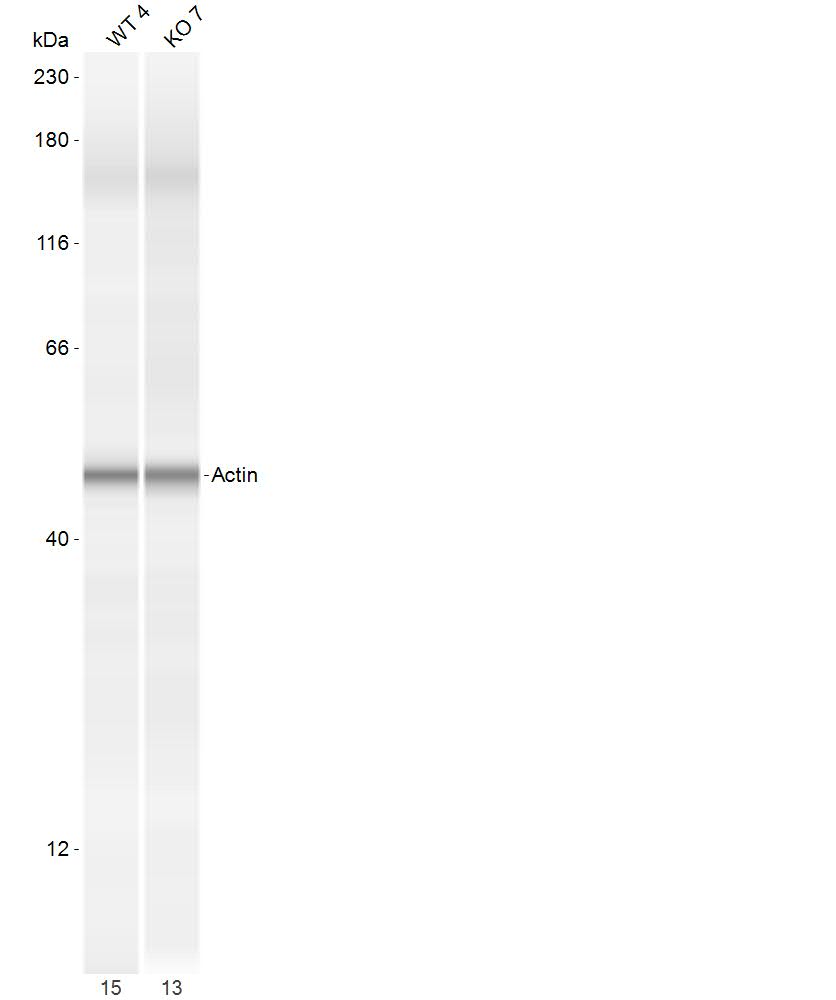

Supplement: Supplementary file 4 — Source data Fig. 3 [file 44319_2024_123_MOESM4_ESM.zip › Figure 3/3G/ActinExpoHDR.jpg]

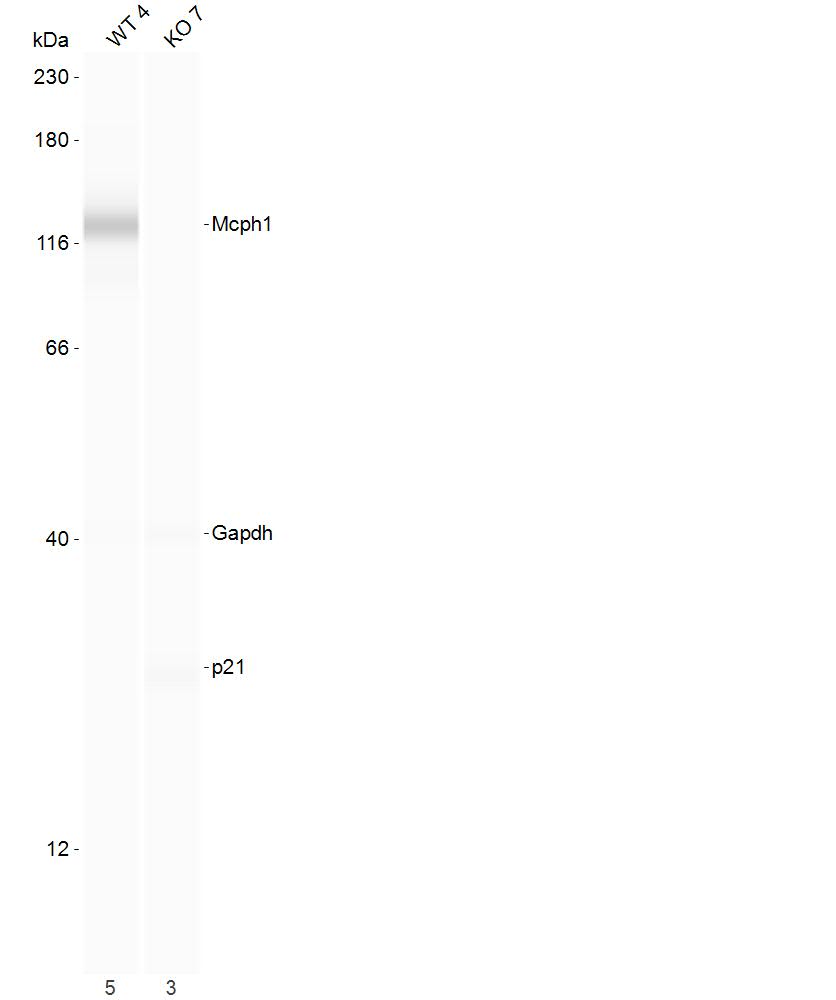

Supplement: Supplementary file 4 — Source data Fig. 3 [file 44319_2024_123_MOESM4_ESM.zip › Figure 3/3G/MCPH1expoHDR.jpg]

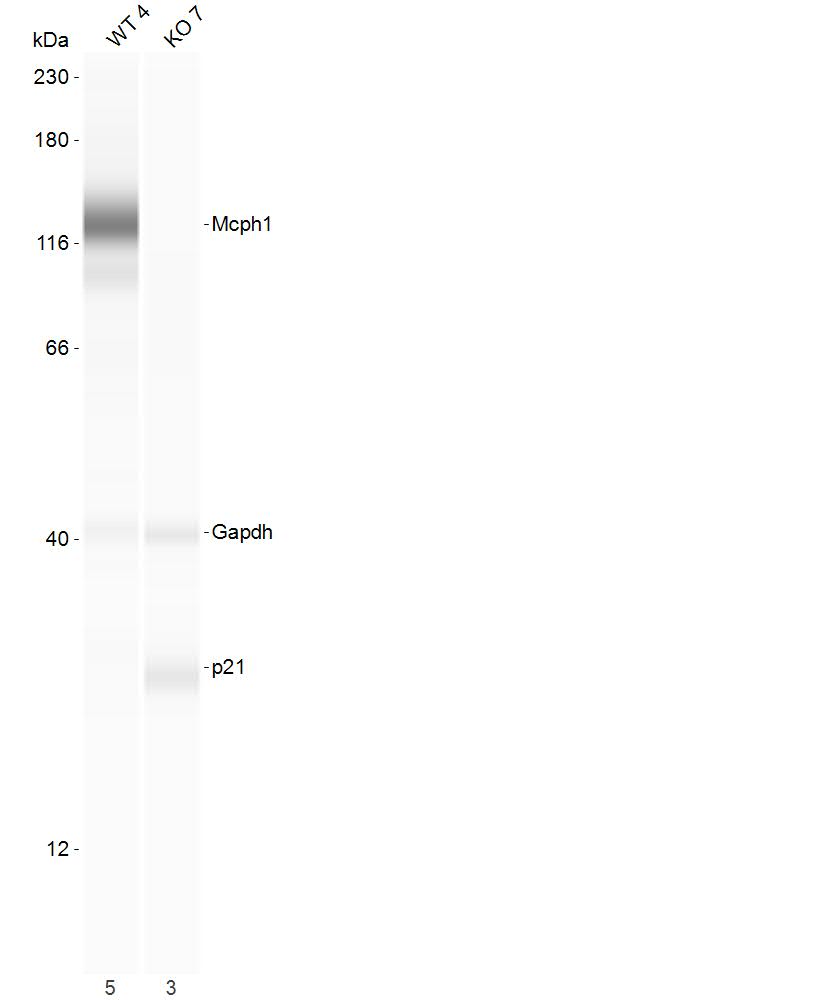

Supplement: Supplementary file 4 — Source data Fig. 3 [file 44319_2024_123_MOESM4_ESM.zip › Figure 3/3G/P21Expo64s.jpg]

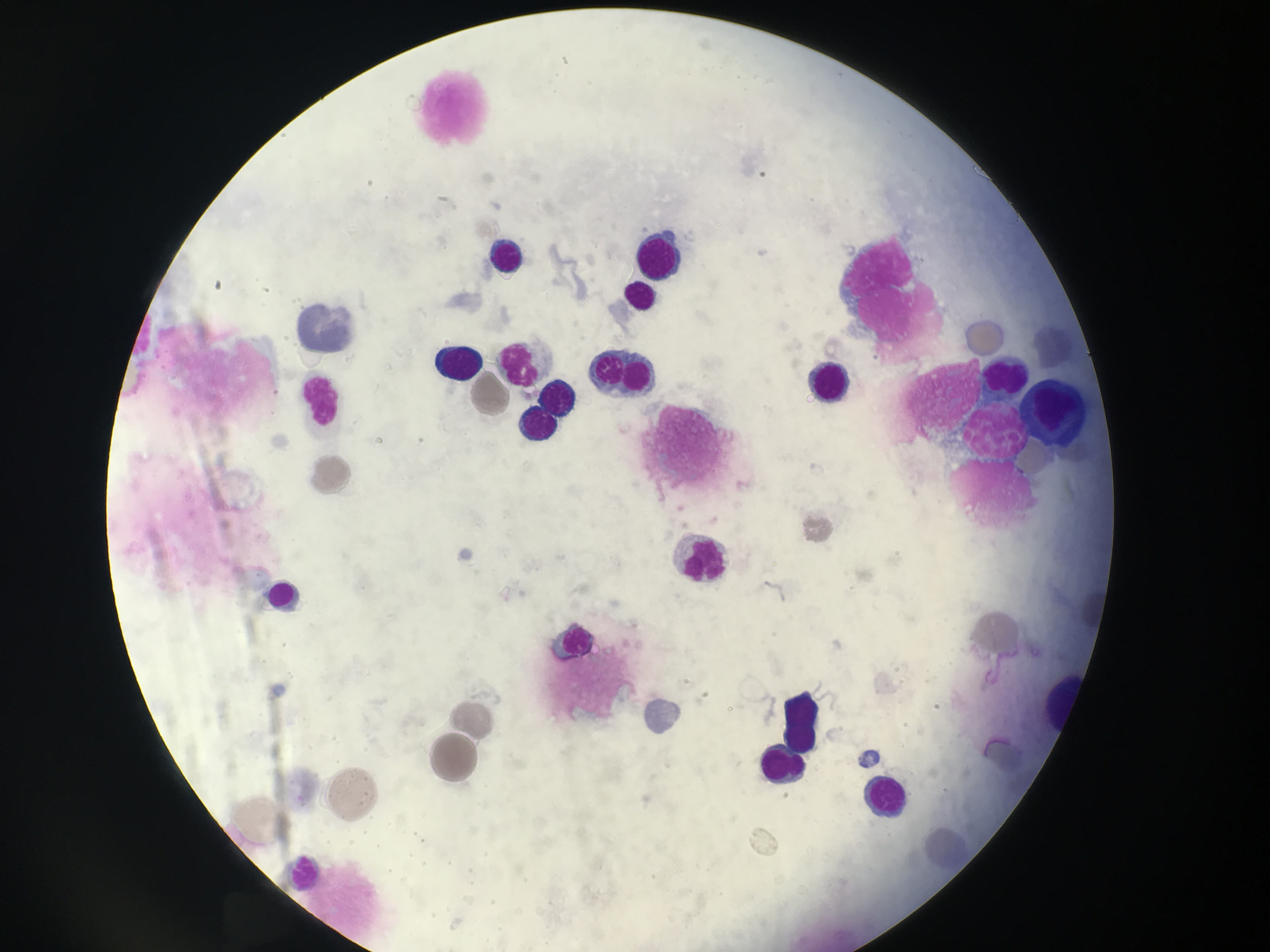

Supplement: Supplementary file 5 — Source data Fig. 4 [file 44319_2024_123_MOESM5_ESM.zip › Figure 4/4E/IMG_2191.JPG]

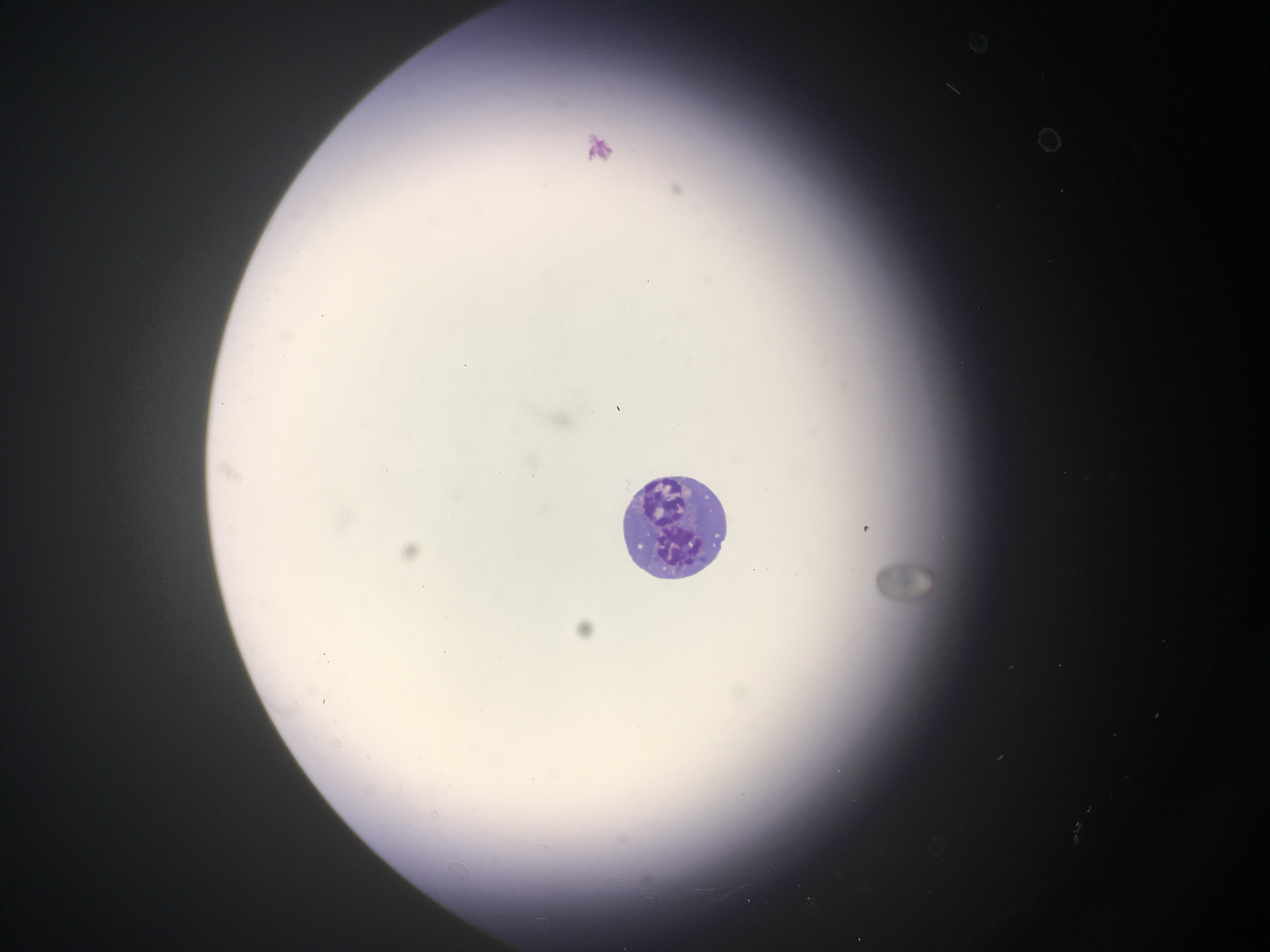

Supplement: Supplementary file 5 — Source data Fig. 4 [file 44319_2024_123_MOESM5_ESM.zip › Figure 4/4F/IMG_1.JPG]

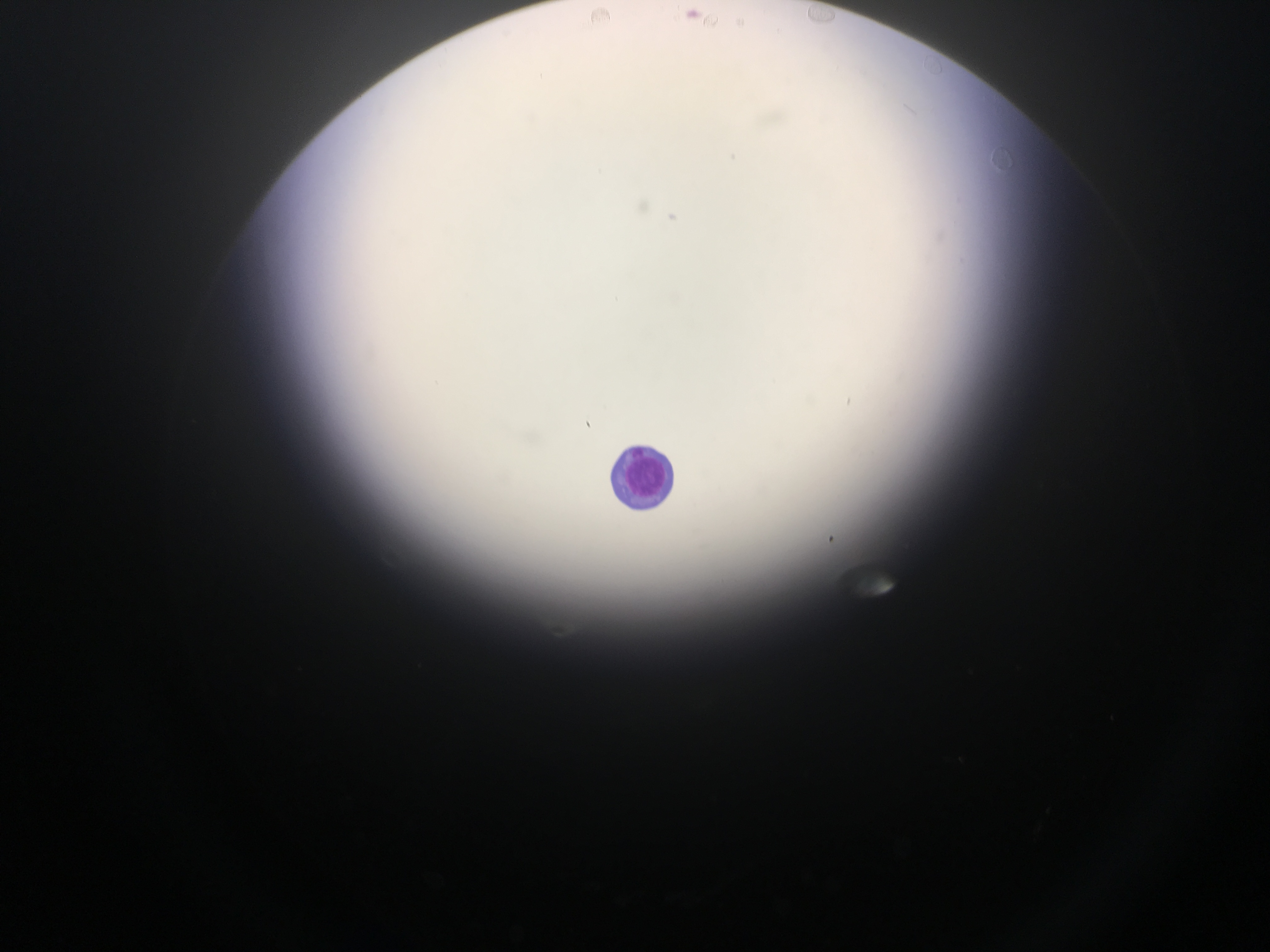

Supplement: Supplementary file 5 — Source data Fig. 4 [file 44319_2024_123_MOESM5_ESM.zip › Figure 4/4F/IMG_2.JPG]

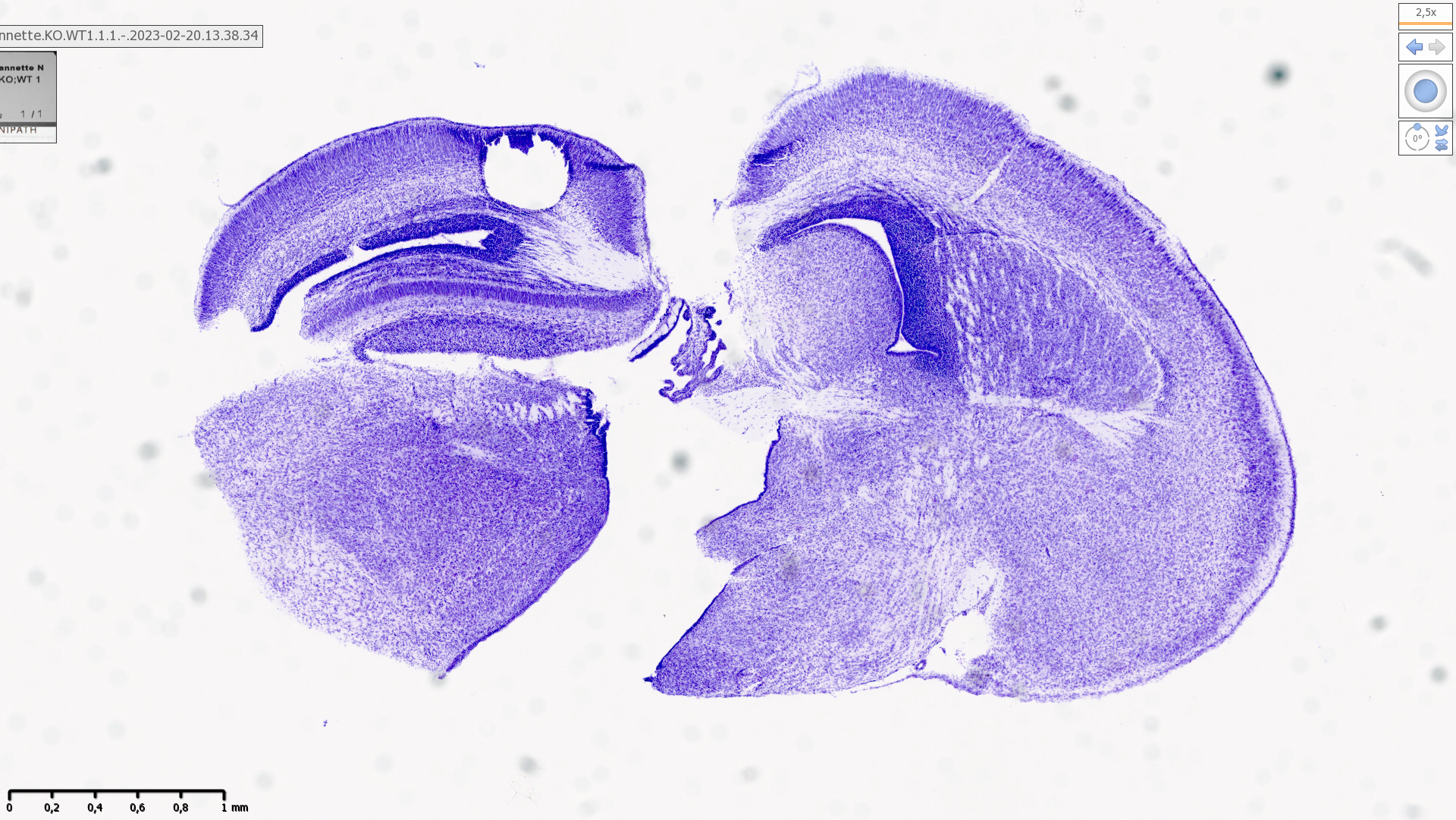

Supplement: Supplementary file 7 — Source data Fig. 6 [file 44319_2024_123_MOESM7_ESM.zip › Figure 6/6A/KOWT2.5x.tif]

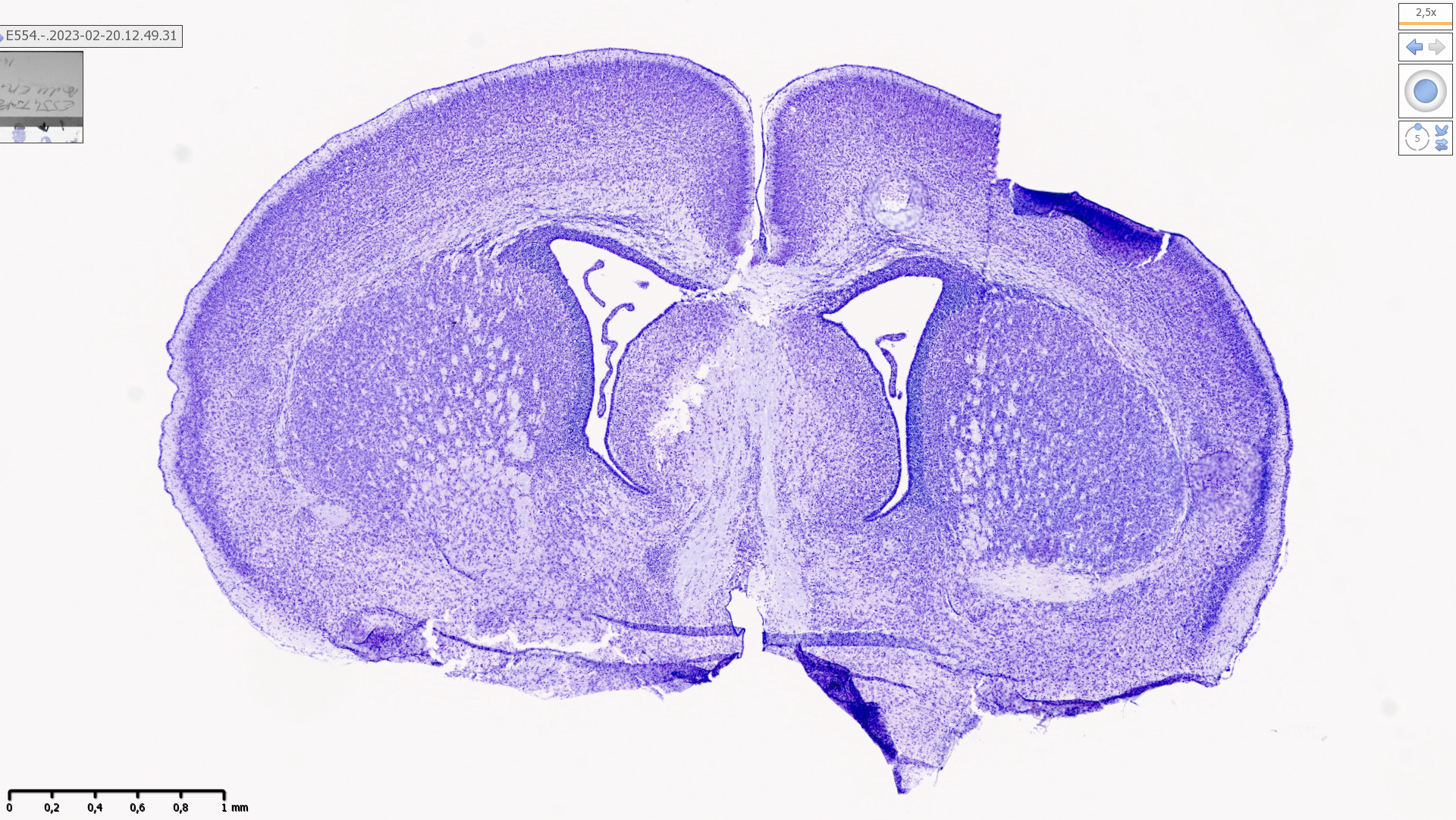

Supplement: Supplementary file 7 — Source data Fig. 6 [file 44319_2024_123_MOESM7_ESM.zip › Figure 6/6A/WTWT2.5x.tif]

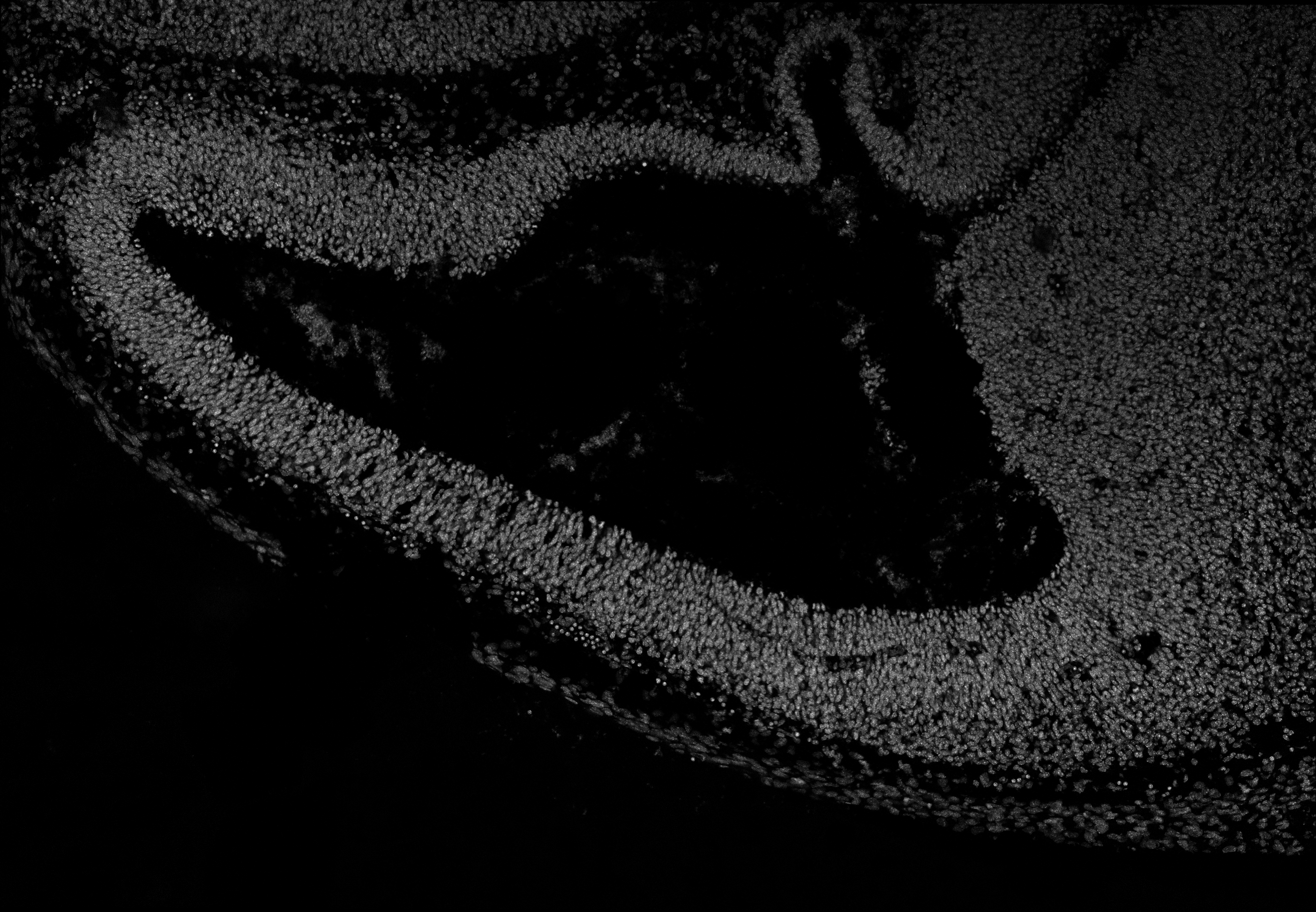

Supplement: Supplementary file 7 — Source data Fig. 6 [file 44319_2024_123_MOESM7_ESM.zip › Figure 6/6E/E12.5 WT3 P21 P16 CD3.tif]
