# Supplementary material for: Mcph1, mutated in primary microcephaly, is also crucial for erythropoiesis
Source: EMBO Rep. 2024 Apr 11;25(5):19. doi: 10.1038/s44319-024-00123-8 (PMC11094029; doi:10.1038/s44319-024-00123-8)
Supplement: Supplementary file 10 — Expanded View Figures [file 44319_2024_123_MOESM10_ESM.pdf]

Expanded View Figures

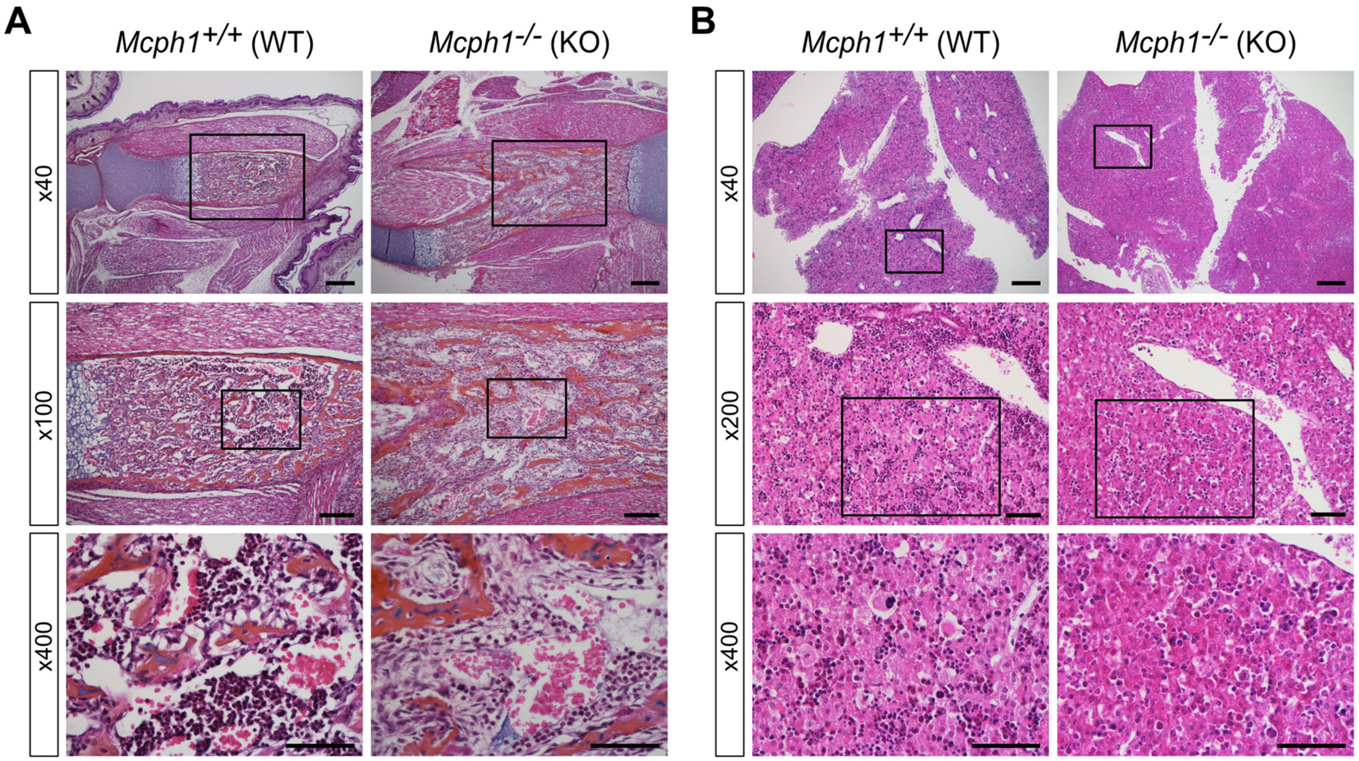

Figure EV1. Hematopoiesis at birth.

Liver and femur from Wild type (WT, *Mcph1*$^{+/+}$) and knockout (KO, *Mcph1*$^{-/-}$) mice were collected at birth (day 0). Hematoxylin and Eosin staining was used to evaluate cellularity on (**A**) femur cross section ×40 (Scale bar = 200 µm), ×100 (Scale bar = 100 µm), ×400 (Scale bar = 50 µm) and (**B**) liver section ×40 (Scale bar = 200 µm), ×200 (Scale bar = 50 µm), ×400 (Scale bar = 50 µm).

© The Author(s)

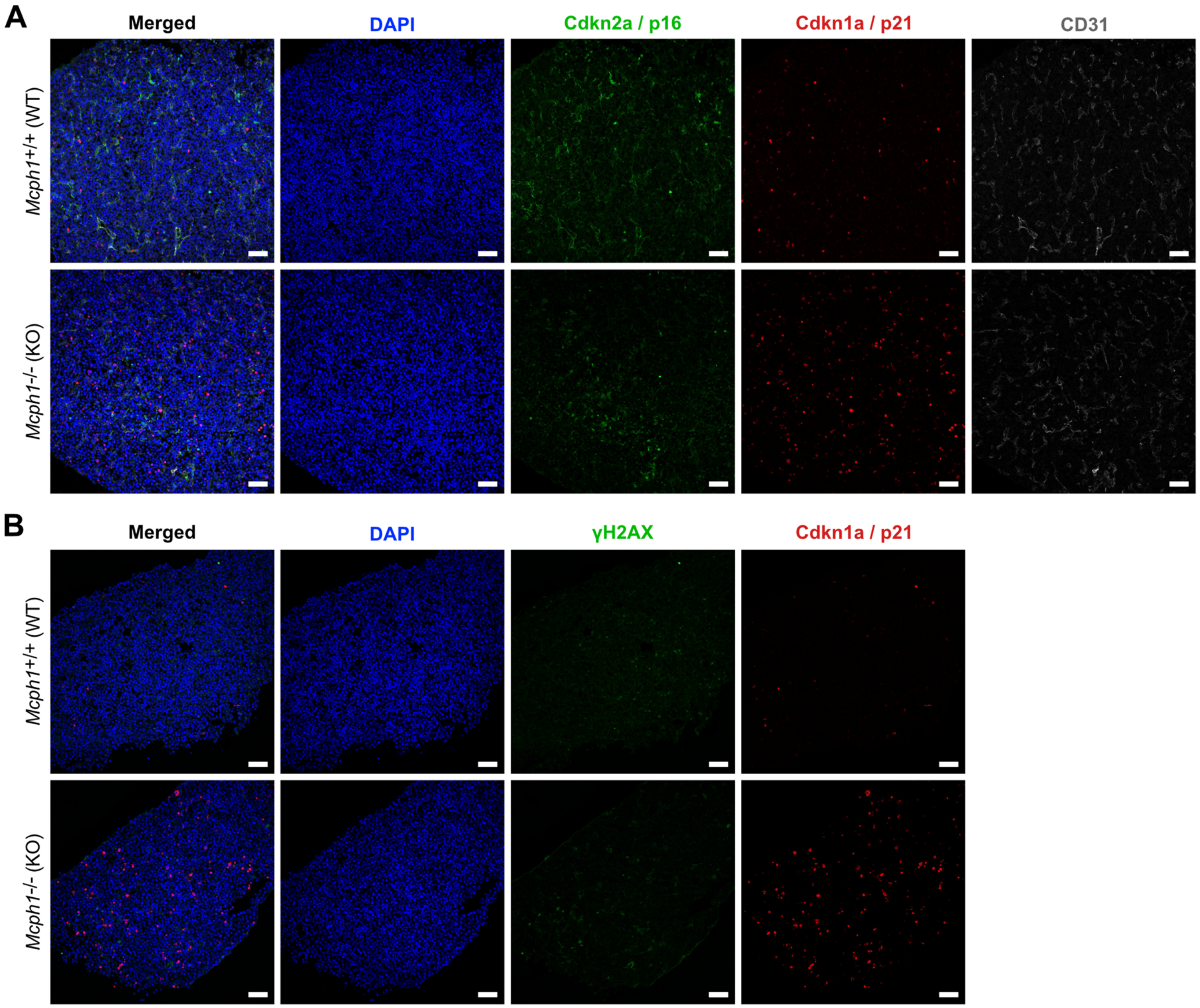

Figure EV2. p21 is overexpressed in fetal liver in the absence of DNA damage and senescence.

Fetal liver sections were obtained from E13.5 embryos of wild type (WT, *Mcph1*$^{+/+}$) and knockout (KO, *Mcph1*$^{-/-}$) mice. (**A**) DAPI-staining (blue) and co-immunofluorescence of p21 (red), p16 (green) and CD31 (gray). (**B**) DAPI-staining (blue) and co-immunofluorescence of p21 (red) and γH2AX (green). Data information: In (**A** and **B**), the scale bars represent 50 μm.

 © The Author(s)

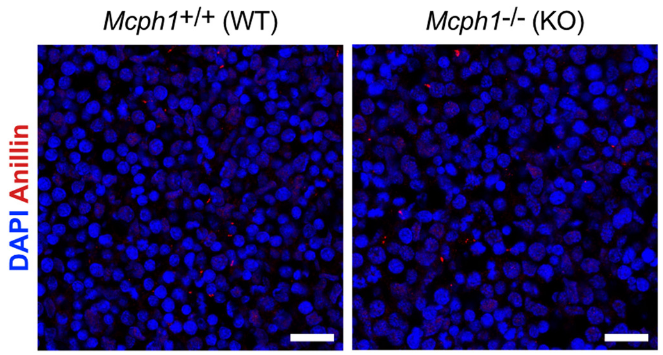

Figure EV3. Anillin, a protein required for the faithfulness of cytokinesis is normally expressed in fetal liver sections.

Fetal liver sections were obtained from E13.5 embryos of wild type (WT, *Mcph1*$^{+/+}$) and knockout (KO, *Mcph1*$^{-/-}$) mice. DAPI-staining (blue) and immunofluorescence of Anillin (red). Scale bars = 20 μm.

© The Author(s)

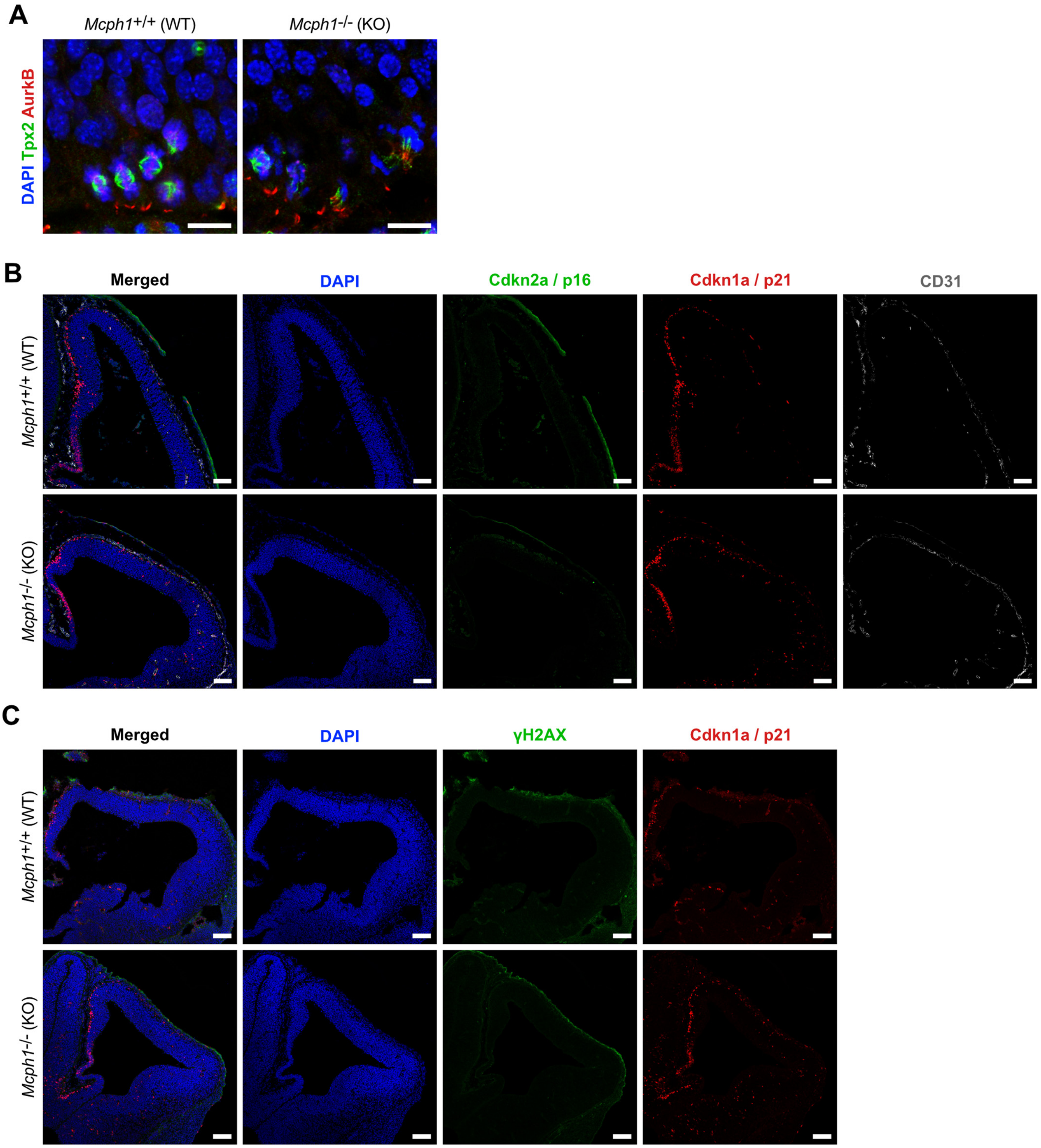

Figure EV4. Cell Division Defect and p21 Overexpression in Fetal Brain Revealed by Immunofluorescence.

Coronal sections were obtained from E12.5 wild type (WT, *Mcph1*$^{+/+}$) and knockout (KO, *Mcph1*$^{-/-}$) mouse embryonic cortex. (**A**) DAPI-staining (blue) and co-IF of Aurora kinase B (AurkB, red) and Tpx2 (green). Scale bar = 10 μm. (**B**) DAPI-staining (blue) and co-immunofluorescence of p21 (red), p16 (green) and CD31 (gray). A magnified view of these images without the p16 layer is shown in Fig. 6E. Scale bar = 100 μm. (**C**) DAPI-staining (blue) and co-immunofluorescence of p21 (red) and γH2AX (green). Scale bar = 100 μm.

 © The Author(s)

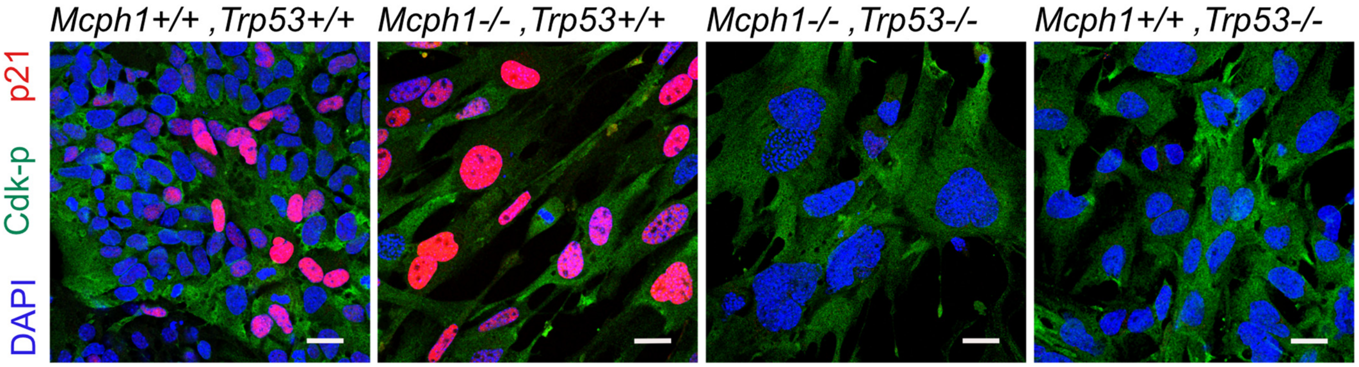

Figure EV5. p21 expression is mediated by p53 in NPC.

Primary cultures of NPCs isolated from *Mcph1*$^{+/+}$,*Trp53*$^{+/+}$; *Mcph1*$^{+/+}$,*Trp53*$^{-/-}$; *Mcph1*$^{-/-}$,*Trp53*$^{+/+}$ and *Mcph1*$^{-/-}$,*Trp53*$^{-/-}$ embryos were stained with DAPI and incubated with p21 and P-Cdk antibodies. Scale bar = 10 µm.
